# Sex Difference in Disease-Related Adverse Events Post-Diagnosis of Lung Cancer Brain Metastases in Medicare Individuals ≥ 66 Years of Age

**DOI:** 10.3390/cancers16172986

**Published:** 2024-08-28

**Authors:** Mantas Dmukauskas, Gino Cioffi, Kristin A. Waite, Aaron G. Mammoser, Andrew E. Sloan, Patrick C. Ma, Jill S. Barnholtz-Sloan

**Affiliations:** 1 Trans Divisional Research Program, Division of Cancer Epidemiology and Genetics, National Cancer Institute, Bethesda, MD 20892, USA; 2 Neuroscience Service Line and Piedmont Brain Tumor Center, Piedmont Health, Atlanta, GA 30309, USAandrew.sloan@piedmont.org (A.E.S.); 3 Penn State Cancer Institute, Penn State Health Milton S. Hershey Medical Center, Penn State College of Medicine, Penn State University, Hershey, PA 17033, USA; patrickma@pennstatehealth.psu.edu; 4 Center for Biomedical Informatics and Information Technology, National Cancer Institute, Rockville, MD 20892, USA

**Keywords:** brain metastases, lung cancer, adverse events, sex differences

## Abstract

**Simple Summary:**

It is known that there are sex differences in adverse events experienced following cancer treatment. However, little is known about sex differences in adverse events experienced in individuals with metastatic cancer. Here, using SEER-Medicare data, we investigate sex differences in adverse events in lung cancer individuals with brain metastasis who are 66 years old and older. Sex differences in adverse events were observed and were dependent on lung cancer histology, age at diagnosis, year of diagnosis and treatment as well as potential interplay between these variables.

**Abstract:**

Sex differences are evident in adverse events (AEs) related to brain tumors, yet sex differences in AEs specific to brain metastases (BrMs) are underexplored. Lung cancer BrMs dominate among BrM, comprising over half of cases. This study examined sex differences in AEs associated with lung cancer BrMs in individuals aged 66 or older using the SEER-Medicare dataset. Multivariable logistic regression, adjusted for demographic factors and comorbidities, stratified by histological subtype, treatment, age, and year of diagnosis were used to analyze AEs among those with BrMs from primary lung tumors. Year of diagnosis was grouped into prior/post-2013, to account for shifts in treatment paradigms. The results showed nuanced sex-specific AEs. Females diagnosed post-2013 with small-cell, squamous-cell, or other non-small-cell carcinoma BrMs had a higher headache likelihood than males. Males with adenocarcinoma post-2013 were more likely to experience brain herniation. Females aged 76 and older with small-cell BrM exhibited increased vision difficulty risk compared to males of the same age, with no significant difference in other age groups. Males treated for adenocarcinoma faced heightened hemorrhagic stroke risk. This study reveals sex-specific disparities in AEs among older individuals with lung cancer BrMs, varying by histological subtype, age, diagnosis year, and treatment.

## 1. Introduction

Lung cancer is one of the most prevalent and deadly forms of cancer worldwide and is responsible for significant morbidity and mortality [1]. Among the sequalae associated with lung cancer, brain metastasis (BrM) is the most significant [2]. Despite advances in treatment of lung cancer and BrM, the prognosis for individuals is poor with a median overall survival of 12 months [3]. Survival rates worsen with advancing age, with individuals aged 65 years and older having a median overall survival of less than 4 months [4,5]. Studies have demonstrated that the associated incidence and outcome of BRM from lung cancer varies with lung cancer histology [6].

Non-small-cell lung cancer (NSCLC), characterized primarily by adenocarcinoma and squamous-cell carcinoma histological subtypes, is the most predominant type of lung cancer, accounting for approximately 80% of lung cancer cases [7]. Other types of NSCLC include large-cell carcinomas and the rarer sarcomatoid carcinoma, adenosquamous carcinoma, and neuroendocrine carcinoma [8]. Among NSCLC individuals, up to 50% will develop BrM [9]. Adenocarcinoma is the most common subtype of NSCLC with a high propensity for BrM. Individuals with adenocarcinoma often present with BrM at initial diagnosis. In contrast, squamous-cell carcinoma is less likely to metastasize to the brain compared to adenocarcinoma; however, these metastases tend to be more aggressive and resistant to therapies. Small-cell lung cancer (SCLC) is highly aggressive with a marked tendency to develop BrM, with BrM presenting early in the course of the disease or after a relapse to treatment [10].

Clinically, BrM from lung cancer presents both diagnostic and therapeutic challenges as symptoms are often non-specific and include headaches, neurological deficits, and cognitive changes, which can significantly decrease quality of life [11]. Treatment for BrM includes surgical resection, radiation therapy, and chemotherapy, including both targeted and immunotherapies. Biomarker testing, particularly for NSCLC, has allowed for the generation of “targeted” therapies, where the chemotherapy selected is directed towards inhibiting the effects of the cancer causing mutation. Currently, there are FDA-approved targeted therapies for lung cancers with mutations and genomic alterations in *EGFR*, *ALK*, *ROS-1*, *NTRK*, *MET*, *RET*, *HER2*, and *BRAF* [10,12,13]. Advances in targeted therapies, particularly after 2013, when the FDA approved *EGFR*-positive NSCLC treatment with Erlotinib, have led to significant improvements in tumor regression response and improved clinical outcomes and survival [14,15]. Each treatment modality, however, has benefits and limitations such as adverse events (AEs), with different risks as compared to disease-related AEs (no treatment) [16,17,18].

Sex differences in the incidence of lung cancer have been well documented [19]. Males have been shown to have higher incidence of lung cancer. This increased incidence has generally been attributed to social behaviors (i.e., smoking history) and environmental exposures [19]. However, a growing body of work suggests that these factors cannot fully account for the observed sex differences in incidence [20]. Further, previous work has identified various risk factors for AE development, in individuals with brain tumors, including comorbidity scores and age, with sex emerging as a noteworthy determinant [21,22,23,24]. While a growing body of work has demonstrated both sex differences in lung cancer incidence as well as AEs in brain tumors, the impact of sex differences on AEs associated with lung cancer BrM has not yet been closely studied. Here, utilizing data from the Surveillance, Epidemiology, and End Results (SEER) Program-Medicare dataset, we aim to investigate and address the impact of sex differences on AEs associated with lung cancer BrM.

## 2. Methods

Study Design and Setting: Individuals diagnosed with primary lung cancer and BrM were obtained from the SEER-Medicare dataset, a population-based data registry that collects demographic, clinical, and cause-of-death information from individuals with cancer covering about 28% of the United States (US) population [25]. Medicare is a US, federally funded health insurance, enrolling primarily individuals that are 65 years and older. Medicare claims are insurance treatment or diagnostic procedure billing data. The National Cancer Institute (NCI) and the Center for Medicare Services (CMS) provide an agreement to link Medicare claims data with the SEER dataset.

Claims Data: The following claims files from the SEER-Medicare dataset were utilized in this study: Medicare Provider Analysis and Review (MedPAR), Carrier Claims (NCH), Durable Medical Equipment (DME), Outpatient Claims, and Medicare Part D Data. The MedPAR file provided data on hospitalization, the NCH and DME files provided information on physician-performed procedures and visits while the Outpatient Claims data contained outpatient procedure information. The Medicare Part D file contained prescription drug data. Each record in these files contained date of service, International Classification of Diseases, ninth/tenth revision, (ICD-9/10) diagnosis codes, Current Procedural Terminology (CPT) procedure codes, or Healthcare Common Procedure Coding System (HCPCS) procedure codes. In this study, claims data files were used to identify date of lung cancer and BrM diagnosis as well as AEs that occurred after the BrM diagnosis.

BrM Identification: SEER provides brain metastasis information only if metastatic disease is synchronous and diagnosed in 2010 or later. Therefore, to include individuals with brain metastasis diagnosed between 2007 and 2010, as well as those that developed non-synchronous BrMs (2007–2017), we followed our previously published method that utilized SEER*Medicare data [26]. BrMs were identified through diagnosis codes for secondary cancer of the central nervous system and procedure codes for brain or head imaging. BrM diagnoses were identified within claims data using ICD9/10 codes 198.3X, C79.3X. In order to ensure the location of metastasis was specifically in the brain, the presence of a head imaging study (CPT codes 70450–70470, 70551–70553, 78607–78608) conducted up to 60 days before or after BrM diagnosis was required [26].

**Figure 1:**
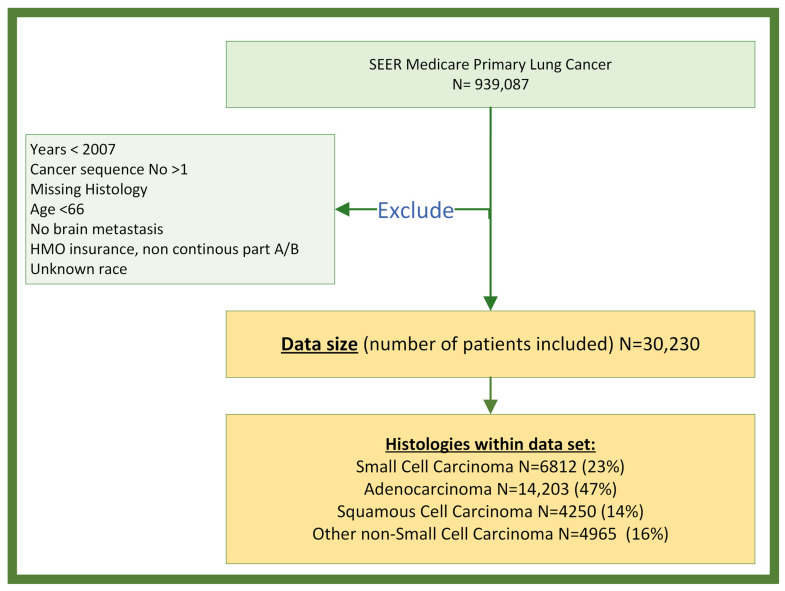
Data selection and exclusion flow chart: the number of individuals selected for the study is summarized.

Inclusion/Exclusion Criteria: From the SEER-Medicare dataset, a total of 30,230 individuals were identified as having a BrM after a lung cancer diagnosis between 2007 and 2017 (Figure 1, described in Results). Only those individuals that were 66 years and older when diagnosed with lung cancer were selected for the study to ensure a full year of comorbidity data extracted from the claims. Individuals were categorized into 4 histopathology groups, either SCLC (ICD-O-3 Codes 8002–8005, 8041–8045), squamous-cell NSCLC (ICD-O-3 Codes 805 [1, 2], 807 [0–6], 8078, 808 [3, 4], 8090, 8094, 8120, 8123), adenocarcinoma NSCLC (ICD-O-3 Codes 8015, 8050, 814 [0, 1, 3–5, 7], 8190, 8201, 8211, 825 [0–5], 8260, 8290, 8310, 832 [0, 3], 8333, 844 [0, 1], 847 [0, 1], 848 [0, 1], 8490, 850 [3, 7], 8550, 857 [0–2, 4, 6]), and other NSCLC (ICD-O-3 Codes 801 [2–4], 8021, 8034, 8082, 8046, 800 [3, 4], 8022, 8030, 803 [1–3, 5], 8120, 8200, 824 [0, 1], 824 [3–6, 9], 8430, 8525, 856 [0, 2], 8575) using International Classification of Disease for Oncology, Third Edition (ICD-O-3) histopathology codes; individuals missing histology codes were excluded. Individuals were excluded if they were additionally enrolled in a Health Maintenance Organization (HMO) insurance coverage or if they were lacking continuous Medicare part A and part B enrollment for 1 year prior to the date of lung cancer diagnosis through the date of death/last follow-up, or the end of adverse event observation time (6–9 months after diagnosis), depending on which occurred first. Individuals with unknown race were also excluded.

Study Variables: BrM treatment information was collected from claims data in the following categories: surgery, radiation, chemotherapy, immunotherapy, and targeted therapy (Supplementary Table S1). Individuals were classified as having treatment if they received any first-line treatment in the first 90 days after BrM diagnosis (Supplementary Figure S1). Individuals whose first-line therapy started more than 90 days after diagnosis were classified as receiving no treatment.

Demographic data collected included sex (male, female), ethnicity (Hispanic, non-Hispanic), age at diagnosis, and race (White, Black, “Other”). The race category “Other” consisted of Native American, Alaskan Indian, Asian, and Pacific Islander, combined due to the insufficient sample size to assess these groups individually. The Elixhauser comorbidity score (0–3, 4–6, 7–9, and >9) was calculated, from the claims data, including all comorbidities 1 year prior to the date of BrM diagnosis with R package ‘Comorbidity 1.0.0’ [27].

Disease-Related Adverse Events (AEs). AEs were identified from the existing literature and the input of three clinicians with expertise in both lung and brain tumors [28,29]. Neurological AEs selected for this study were as follows: epilepsy, ischemic stroke, hemorrhagic stroke, vasogenic edema, brain herniation, hydrocephalus, neurological deficit, headaches, vision impairment, paralysis, and leptomeningeal disease (Supplementary Table S2). These complications were delineated as binary outcomes (present/absent) if they manifested between the start of treatment or in case of no treatment from the date of BrM diagnosis up to 180 days thereafter (present) or were absent during this time period (absent).

Statistical Analysis: Statistical analyses were conducted using R 4.1 software. Descriptive statistics, stratified by sex, were assessed to show differences in demographic and clinical factors. Continuous data were assessed using *t*-tests and categorical data were assessed with either Fisher’s exact test or Pearson’s Chi-squared test where appropriate. The male/female odds ratio (M/F OR) and 95% confidence intervals [95% CI] of experiencing an AE were calculated using multivariable logistic regression, adjusted for demographic variables and Elixhauser comorbidity score. Each AE category within every histological subtype was analyzed separately. Distinct analyses were executed for each histological subgroup and criteria of stratification. There were 3 separate assessment categories used in this study: age, receipt of treatment, and year of diagnosis. Individuals were split into 3 age groups of 66–70, 71–75, and 75+ years with the purpose of having a similar share of individuals between the 3 groups. Receipt of treatment was defined as either receiving no BrM treatment or receiving any type of BrM treatment (surgery, radiation, chemotherapy) (Supplementary Table S1). For year of diagnosis, individuals were grouped into 2 groups based upon year of diagnosis (2007–2013 or 2014–2017). The year 2013 was chosen as the dividing point, as after 2013, there was an increase in the number of FDA-approved targeted/immunotherapies therapies and changes in patterns of care (Supplementary Figure S2) [30,31,32,33]. Counts were suppressed when fewer than 11 occurrences were reported within a cell, or where the inclusion of the count would allow for back-calculation of the suppressed values. Suppressed cases are included in the total counts for a given category. Statistical significance was set at a threshold of *p* < 0.05.

## 3. Results

Study Population: The SEER-Medicare dataset contained 939,087 individuals diagnosed with primary lung cancer between 1999 and 2017. A total of 908,857 individuals were excluded for at least one of the following criteria: year of diagnosis occurring before 2007, diagnosis of non-first-sequence tumor, age of diagnosis <66 years, lack of BrM, a record of HMO or non-continuous part A and part B enrollment, or unknown race (Figure 1). The final dataset, consisting of individuals diagnosed with lung cancer BrM, encompassed a total of 30,230 subjects, comprising 14,541 (48%) males and 15,689 (52%) females. There were 6812 (23%) diagnosed with SCLC, 14,203 (47%) with adenocarcinoma NSCLC, 4250 (14%) with squamous-cell NSCLC, and 4965 (16%) with other NSCLC (Table 1). Overall, the majority were white (87%) and non-Hispanic (96%). Median ages at diagnosis for both male and female were 73 years old.

There were 10,737 (35%) individuals diagnosed between an age of 66 and 70, 8957 (30%) between an age of 71 and 75, and 10,536 (35%) aged ≥76. A total of 17,675 (58%) BrM were synchronous, with 12,555 (42%) occurring asynchronously. There were 17,452 (58%) individuals diagnosed before 2007–2013, and 12,778 (42%) diagnosed between 2014 and 2017. A total of 14,814 (49%) received treatment within 3 months after BrM diagnosis, whereas 15,416 (51%) received no treatment.

**Figure 2:**
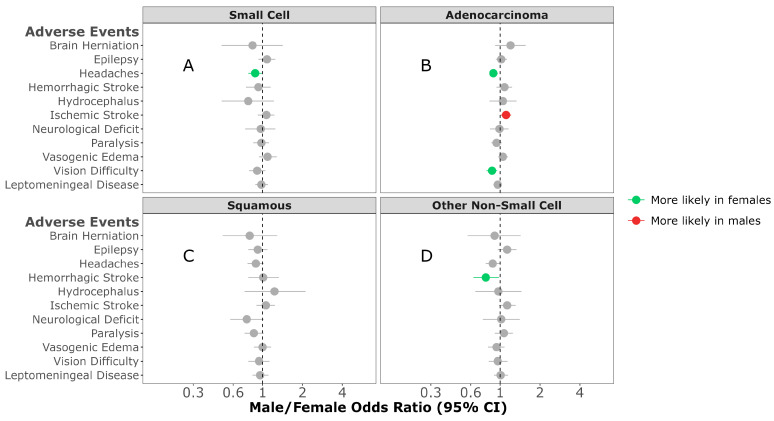
Male/female odds ratio of adverse events for individuals with small-cell (**A**), adenocarcinoma (**B**), squamous-cell carcinoma (**C**), or other non-small-cell carcinoma (**D**) BrM OR calculated using multivariable logistic regression model. All groups were adjusted for race, ethnicity, time to BrM after initial cancer diagnosis, Elixhauser score, year of diagnosis, age at BrM diagnosis, surgery, radiation, chemotherapy, targeted therapy, and immunotherapy categories.

Overall difference in AEs: In individuals diagnosed with SCLC BrM or adenocarcinoma BrM, females were more likely to experience headaches (M/F OR = 0.88; 95% CI, 0.78–0.99, *p* = 0.029; M/F OR = 0.89; 95% CI, 0.83–0.96, *p* = 0.003, respectively) (Figure 2A,B). Males diagnosed with adenocarcinoma BrM were more likely to experience ischemic stroke (M/F OR = 1.11; 95% CI, 1.01–1.21, *p* = 0.022) (Figure 2B). Females diagnosed with other NSCLC were more likely to develop hemorrhagic stroke (M/F OR = 0.78; 95% CI, 0.63–0.98, *p* = 0.029) (Figure 2D). No sex difference was observed in any of the AEs developed in individuals diagnosed with squamous-cell carcinoma (Figure 2C).

**Figure 3:**
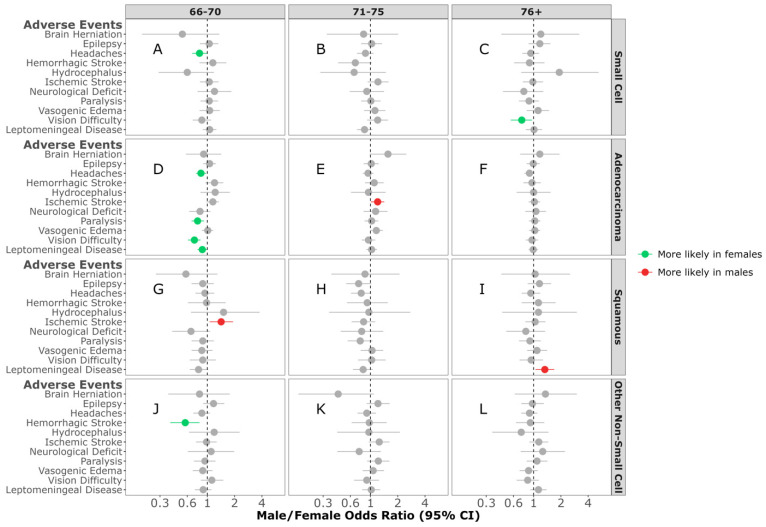
Male/female odds ratio of adverse events for individuals with small-cell BrM aged 66–70 (**A**), 71–75 (**B**), 76+ (**C**), adenocarcinoma BrM aged 66–70 (**D**), 71–75 (**E**), 76+ (**F**), squamous-cell carcinoma BrM aged 66–70 (**G**), 71–75 (**H**), 76+ (**I**), or other non-small-cell carcinoma BrM aged 66–70 (**J**), 71–75 (**K**), 76+ (**L**). OR calculated using multivariable logistic regression model. All groups were adjusted for race, ethnicity, time to BrM after initial cancer diagnosis, Elixhauser score, year of diagnosis, surgery, radiation, chemotherapy, targeted therapy, and immunotherapy categories.

Age Difference in AEs: In individuals diagnosed between 66 and 70 years of age with SCLC BrM, females were more likely to experience headaches (M/F OR = 0.82; 95% CI, 0.68–0.99, *p* = 0.038) (Figure 3A). No sex differences were observed in any of the AEs between individuals diagnosed with SCLC BrM between 71 and 75 years of age (Figure 3B). Females diagnosed with SCLC BrM who were ≥76 years of age were more likely to develop vision difficulty (M/F OR = 0.73; 95% CI, 0.56–0.95, *p* = 0.022) when compared to males (Figure 3C). In individuals diagnosed between 66 and 70 years of age with adenocarcinoma NSCLC BrM, females were more likely to experience headaches (M/F OR = 0.86; 95% CI, 0.76–0.98, *p* = 0.02), paralysis (M/F OR = 0.78; 95% CI, 0.67–0.92, *p* = 0.002), vision difficulty (M/F OR = 0.71; 95% CI, 0.60–0.83, *p* < 0.001), or develop a leptomeningeal disease (M/F OR = 0.88; 95% CI, 0.78–1.00, *p* = 0.043) compared to males (Figure 3D). In contrast, males aged 71–75 years of age were more likely to experience an ischemic stroke (M/F OR = 1.22; 95% CI, 1.03–1.44, *p* = 0.024) (Figure 3E). No sex differences were observed in any of the AEs between individuals diagnosed with adenocarcinoma BrM ≥ 76 of age (Figure 3F). In individuals diagnosed with squamous NSCLC BrM, males aged 66–70 years old were more likely to experience an ischemic stroke when compared to females (M/F OR = 1.43; 95% CI, 1.06–1.93, *p* = 0.02) (Figure 3G). No sex differences were observed in any of the AEs in individuals aged 71–75 years at diagnosis (Figure 3H). Those aged ≥76 years of age were more likely to experience leptomeningeal disease when compared to females (M/F OR = 1.32; 95% CI, 1.04–1.68, *p* = 0.022) (Figure 3I). Among those diagnosed with other NSCL BrM, females aged 66–70 years were more likely to develop hemorrhagic stroke (M/F OR = 0.57; 95% CI, 0.39–0.82, *p* = 0.003) (Figure 3J). No sex differences were observed in any of the AEs in individuals diagnosed with other NSCL BrM aged 71–75 (Figure 3K) or ≥76 years of age (Figure 3L).

**Figure 4:**
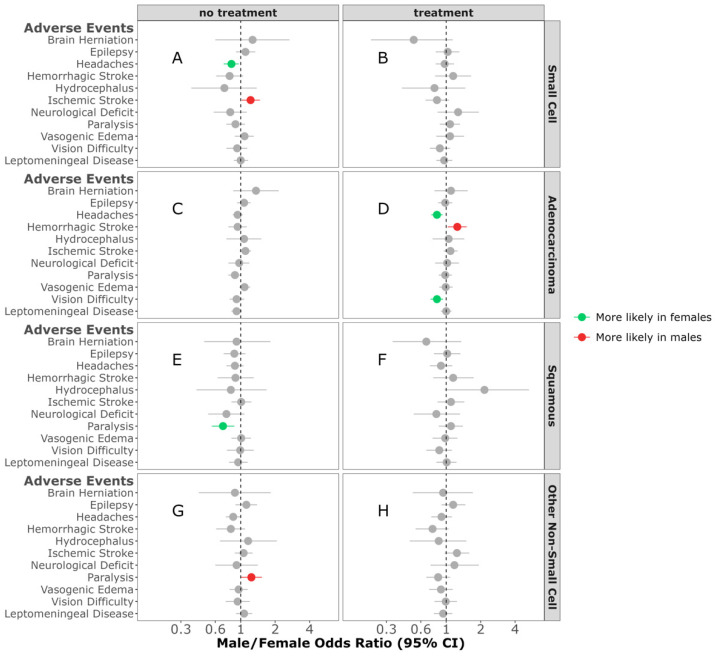
Male/female odds ratio of adverse events for individuals with small-cell BrM receiving treatment (**A**) or no treatment (**B**), adenocarcinoma BrM receiving treatment (**C**) or no treatment (**D**), squamous-cell carcinoma BrM receiving treatment (**E**) or no treatment (**F**), or other non-small-cell carcinoma BrM receiving treatment (**G**) or no treatment (**H**). OR calculated using multivariable logistic regression model. All groups were adjusted for race, ethnicity, time to BrM after initial cancer diagnosis, Elixhauser score, year of diagnosis, and age at diagnosis. Additionally, the treatment groups were adjusted for surgery, radiation, chemotherapy, targeted therapy, and immunotherapy categories.

Cancer-Directed Treatment Difference in AEs: In individuals diagnosed with SCLC BrM who received no treatment, females were more likely to have headaches (M/F OR = 0.83; 95% CI, 0.71–0.96, *p* = 0.012) (Figure 4A). No sex differences were observed in any of the AEs in individuals diagnosed with SCLC BrM who received treatment (Figure 4B). No sex differences were observed in any of the AEs between individuals diagnosed with adenocarcinoma BrM who did not receive treatment (Figure 4C). Females, compared to males, were more likely to have headaches in those diagnosed with adenocarcinoma NSCLC BrM, and received treatment (M/F OR = 0.82; 95% CI, 0.73–0.92, *p* < 0.001) (Figure 4D). In individuals who received no treatment for their squamous NSCLC BrM, females were more likely to be diagnosed with paralysis when compared to males (M/F OR = 0.70; 95% CI, 0.56–0.88, *p* = 0.002) (Figure 4E). No sex difference was observed in any of the AEs in individuals receiving treatment for squamous NSCL BrM (Figure 4F). In contrast, in individuals diagnosed with other NSCLC BrM who did not receive treatment, paralysis was more likely in males compared to females (M/F OR = 1.25; 95% CI, 1.02–1.54, *p* = 0.034) (Figure 4G). No sex differences were observed in any of the AEs between individuals diagnosed with other NSCL BrM and receiving treatment (Figure 4H).

**Figure 5:**
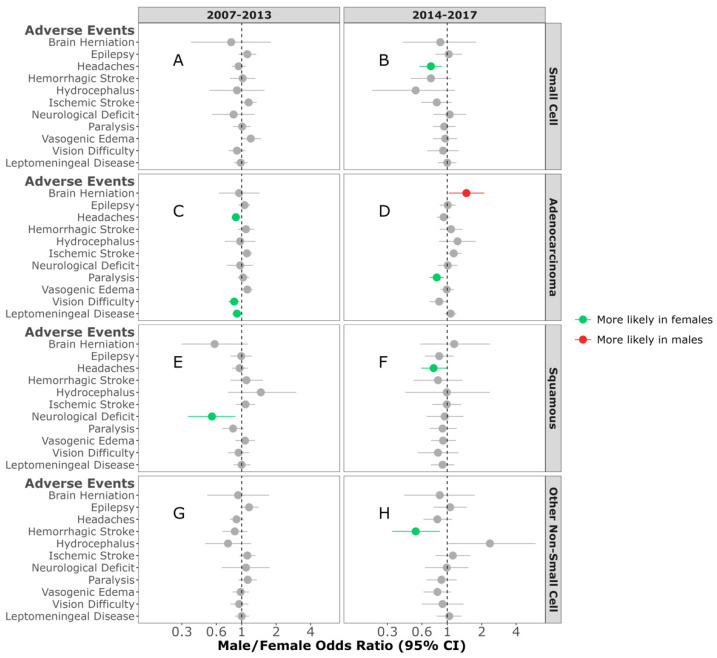
Male/female odds ratio of adverse events for individuals with small-cell BrM diagnosed between 2007–2013 (**A**) and 2014–2017 (**B**), adenocarcinoma BrM diagnosed between 2007–2013 (**C**) and 2014–2017 (**D**), squamous-cell carcinoma BrM diagnosed between 2007–2013 (**E**) and 2014–2017 (**F**), or other non-small-cell carcinoma BrM diagnosed between 2007–2013 (**G**) and 2014–2017 (**H**). OR calculated using multivariable logistic regression model. All groups were adjusted for race, ethnicity, time to BrM after initial cancer diagnosis, Elixhauser score, age at diagnosis, surgery, radiation, and chemotherapy categories.

In an effort to assess the impact of the increased availability of FDA-approved targeted therapies after 2013, we assessed AE pre- and post-2013 (2007–2013 and 2014–2017). In those individuals diagnosed between 2014 and 2017, females diagnosed with either SCLC BrM or squamous NSCLC BrM were more likely to experience headaches (M/F OR = 0.72; 95% CI, 0.57–0.9, *p* = 0.005, (M/F OR = 0.76; 95% CI, 0.58–0.99, *p* = 0.041, respectively) (Figure 5B,F). In contrast, in those diagnosed between 2007 and 2013 with adenocarcinoma NSCLC BrM, females were more likely to experience headaches compared to males (M/F OR = 0.89; 95% CI, 0.81–0.98, *p* = 0.012) (Figure 5C). No sex difference was observed in any of the AEs between individuals diagnosed with SCLC BrM and other NSCL BrM between 2007 and 2013 (Figure 5A,G).

## 4. Discussion

This, to our knowledge, is the first descriptive epidemiology analysis of AEs in lung BrM. The analysis demonstrates that there are sex differences in the AEs experienced in lung cancer individuals diagnosed with BrM, which depend on the type of lung cancer. Additionally, these sex differences in AEs may be influenced by age and treatment.

Previous work has demonstrated that the likelihood of experiencing an AE increases with age until 60 years when it then plateaus [34]. We found most differences in the AEs experienced between males and females occurred at the younger ages included in our study. The proportion of individuals between the 71 and 75 and ≥76-year-old age groups was similar, and changes in the number of AEs were minimal, which may suggest that there are fewer sex differences at older ages. It would be interesting to examine if there are greater differences between male and females in ages younger than 66; however, this is beyond the scope of this analysis and dataset. One may not expect large differences in AE profiles between males and females at older ages as the impact of sex hormone pathways is generally thought to decrease between the sexes as age increases [35]. This is generally thought to be due to females going through menopause as well as males having a decrease in testosterone levels [36,37]. The sex differences in AEs observed here suggest that these AEs are unlikely to be impacted by sex hormone signaling, which is not expected to be different between males and females at our selected age group. Additionally, recent studies have suggested that sex differences in cancer incidence cannot be solely due to sex hormone pathways [38]; rather, these differences may be due to sex differences in other biologic pathways. Our analysis here provides additional evidence to support this. Differences were also observed between different types of lung cancer, which may indicate that other biological pathways, outside of sex hormone pathways, contribute to the sex differences in AEs observed in this study. If sex hormone pathways were solely impacting an AE, one might expect to see that specific AE in all types of lung cancer. However, we found that there were differences in the AEs amongst all the histopathologies studied here.

Previous studies have demonstrated that leptomeningeal disease, while more common in those over 60 years old, manifests to an equal degree in both NSCLC and SCLC with no notable sex differences [39,40,41]. However, here, females were more likely to have leptomeningeal disease when diagnosed with adenocarcinoma NSCLC BrM between 66 and 70 years of age while males were more likely to have leptomeningeal disease when diagnosed ≥ 76 years with squamous NSCLC, potentially indicating a unique mechanism between these two different histology groups.

The analysis here suggests that there are sex differences in AEs, which may be dependent upon receipt of treatment. BrM treatment is often recommended as untreated metastatic disease has a limited survival prognosis of only one to two months [42]. Despite this, treatment-dependent AEs also have the potential to profoundly alter the quality of life. Consequently, a substantial amount of individuals opt for no treatment, reflecting the nuanced balance between treatment benefits and associated risks [43]. Indeed, in this study, approximately 51% of individuals did not receive treatment for their BrM. While existing therapies encompassing surgical intervention and radiation therapy are frequently recommended, their efficacy in terms of prognosis remains variable [42]. Every therapeutic modality is accompanied by toxicities and a risk of experiencing an AE with the likelihood and severity of these AEs contingent upon many factors.

The advent of targeted therapies began with EGFR-targeted treatments for lung cancer and changed the outlook for lung cancer dramatically. The discovery and development of additional therapeutic targets for lung cancer has dramatically accelerated since 2013, with the approval of therapies targeted towards *EGFR* mutations, *ALK*-, *ROS*-1-, *RET*-, *NTRK*-1/2/3-fusions, *METex14*, *BRAF* and *KRAS* mutations, as well as PD-1/PD-L1 and CTLA-4 [9,44,45,46], which have their own respective unique AE risks [47].

In our study, sex-based differences were observed between different histology and treatment profiles, suggesting that these AE profiles may be dependent on a potential interplay between the two. For example, paralysis has a different sex difference profile depending on the histology and treatment. The cerebellum is responsible for movement and coordination; thus, metastases to this area, which occur in >50% of individuals with lung cancer BrM, may result in paralysis or other movement-related AEs that greatly affect the quality of life for individuals with BrM [48,49]. Although we could not assess the proportion of individuals with BrM in the cerebellum, the results observed here suggest that there is a sex-dependent interplay between histology and treatment that may contribute to the observed sex differences in paralysis.

Some AEs are known to be more likely to be present in one sex compared to the other and we have observed similar trends in our study. Headaches are more common among females diagnosed with brain tumors [50,51]. While females were more likely to experience headaches in this analysis, it was dependent upon the type of histology as well as treatment. It is generally thought that the sex differences observed in headache prevalence are due to the impact of sex hormones. However, given the age of individuals in this study, one may expect that the role of sex hormone pathways would be minimal. This may suggest that the treatments may be impacting other pathways that modulate headaches. Finally, headaches are non-serious AEs, due to which care is not always sought, resulting in underreported cases [52].

There are several limitations inherent to this study. Firstly, the findings derived from this investigation may not be universally applicable across all ages, as both the onset of lung cancer and subsequent BrM can manifest across a broad age spectrum. Therefore, the generalizability of our results is limited to older adults within the US. Secondly, the utilization of Medicare claims data imposes constraints, beyond age, precluding the comprehensive identification of procedures covered under private insurance plans. This limitation introduces the potential for bias, particularly in scenarios where individuals maintain dual insurance coverage, with employer-sponsored insurance serving as the primary insurer and Medicare as the secondary provider. Consequently, claims would be initially routed to the primary insurer, thereby omitting information from the Medicare claims dataset. Additionally, Medicare lacks the retrospective collection of data predating Medicare enrollment, thereby limiting the availability of comorbidity data beyond a one-year timeframe preceding the diagnosis. This constraint poses potential limitations on the comprehensiveness of comorbidity data captured in the analysis.

Due to the data being stratified by multiple factors, caution is warranted due to the number of tests being performed affecting the confidence intervals and potentially resulting in 5% of the significant findings being overinterpretations of limited data. Hence, more work is needed to validate these results in other cohorts. Furthermore, this study took a broad approach by assessing the impact of any treatment, without considering the specific effects that individual medication types or treatment sequencing may possess. Even though it is possible to extract specific treatment modalities from the claims data, the number of individuals per treatment was not sufficient to perform robust analysis to extract meaningful information. This omission may overlook nuanced differences in AE profiles associated with distinct therapeutic interventions. Additionally, both SEER and Medicare claims data do not contain information on biomarkers, such as *KRAS*, *EGFR*, or *ALK* mutation status. Lastly, accuracy of CNS metastases diagnosis and imaging procedure codes for detecting BrM is a limitation of this study, and careful consideration should be given to the use of these data.

## 5. Conclusions

BrM represents a formidable complication of lung cancer, impacting both individual survival and quality of life. Advances in treatment should also consider the impact an AE may have on the quality of life. This study is the first to analyze sex differences in AEs in a large lung cancer BrM dataset. Our findings of sex differences in AE profiles in these cancers highlight the need for additional research to evaluate additional factors and to elucidate the mechanisms and implications of the observed differences. Further, it demonstrates the need for potential sex differences in AEs to be taken into consideration in the treatment of individuals aged 66 years and older diagnosed with lung cancer BrM. These differences manifest across histological subtypes and various stratifications, including age cohorts and treatment. It is important for healthcare providers to incorporate an awareness of sex differences into treatment discussions and plans for older individuals aged 66 and above diagnosed with lung cancer BrM. By recognizing and addressing these differences, healthcare professionals can strive towards more equitable and effective care delivery, ultimately optimizing individual outcomes and quality of life. Continued research will be necessary so that therapeutic approaches can be refined for individuals.

## Figures and Tables

**Table 1:** Descriptive statistics of demographic and clinical factors of individuals with lung cancer BrM. The data are stratified by lung cancer histology and sex.

	Small-Cell			Adenocarcinoma			Squamous			Other Non-Small-Cell		
	Female, *n* = 3646 (54%) ^1^	Male, *n* = 3166 (46%) ^1^	*p*-Value ^2^	Female, *n* = 7860 (55%) ^1^	Male, *n* = 6343 (45%) ^1^	*p*-Value ^2^	Female, *n* = 1760 (41%) ^1^	Male, *n* = 2490 (59%) ^1^	*p*-Value ^2^	Female, *n* = 2423 (49%) ^1^	Male, *n* = 2542 (51%) ^1^	*p*-Value ^2^
Race			<0.001			0.016			0.4			0.073
White	3340 (92%)	2815 (89%)		6745 (86%)	5351 (84%)		1530 (87%)	2140 (86%)		2154 (89%)	2217 (87%)	
Non-White	306 (8.4%)	351 (11%)		1115 (14%)	992 (16%)		230 (13%)	350 (14%)		269 (11%)	325 (13%)	
Unknown	0 (0%)	0 (0%)		0 (0%)	0 (0%)		0 (0%)	0 (0%)		0 (0%)	0 (0%)	
Ethnicity			0.3			0.4			0.018			0.023
Non-Hispanic	3533 (97%)	3052 (96%)		7513 (96%)	6045 (95%)		1694 (96%)	2358 (95%)		2328 (96%)	2408 (95%)	
Hispanic	113 (3.1%)	114 (3.6%)		347 (4.4%)	298 (4.7%)		66 (3.8%)	132 (5.3%)		95 (3.9%)	134 (5.3%)	
Elixhauser Score			0.014			0.15			0.2			0.12
0–3	956 (26%)	882 (28%)		3084 (39%)	2435 (38%)		522 (30%)	804 (32%)		857 (35%)	971 (38%)	
4–6	1397 (38%)	1275 (40%)		3067 (39%)	2447 (39%)		737 (42%)	980 (39%)		975 (40%)	964 (38%)	
7–9	941 (26%)	751 (24%)		1345 (17%)	1120 (18%)		359 (20%)	525 (21%)		453 (19%)	448 (18%)	
10+	352 (9.7%)	258 (8.1%)		364 (4.6%)	341 (5.4%)		142 (8.1%)	181 (7.3%)		138 (5.7%)	159 (6.3%)	
Age			0.15			<0.001			<0.001			0.024
66–70	1392 (38%)	1261 (40%)		2626 (33%)	2277 (36%)		551 (31%)	918 (37%)		790 (33%)	922 (36%)	
71–75	1133 (31%)	998 (32%)		2257 (29%)	1859 (29%)		507 (29%)	738 (30%)		732 (30%)	733 (29%)	
76+	1121 (31%)	907 (29%)		2977 (38%)	2207 (35%)		702 (40%)	834 (33%)		901 (37%)	887 (35%)	
Treatment			0.6			0.5			0.14			0.7
No Treatment	1929 (53%)	1654 (52%)		3809 (48%)	3036 (48%)		969 (55%)	1314 (53%)		1313 (54%)	1392 (55%)	
Treatment	1717 (47%)	1512 (48%)		4051 (52%)	3307 (52%)		791 (45%)	1176 (47%)		1110 (46%)	1150 (45%)	
Year of Diagnosis			0.068			>0.9			0.6			0.4
2007–2013	2401 (66%)	2151 (68%)		4965 (63%)	4006 (63%)		1179 (67%)	1647 (66%)		1846 (76%)	1961 (77%)	
2014–2017	1245 (34%)	1015 (32%)		2895 (37%)	2337 (37%)		581 (33%)	843 (34%)		577 (24%)	581 (23%)	
Synchronous Metastases			0.006			<0.001			0.4			<0.001
Asynchronous	1820 (50%)	1475 (47%)		3255 (41%)	2354 (37%)		788 (45%)	1085 (44%)		926 (38%)	852 (34%)	
Synchronous	1826 (50%)	1691 (53%)		4605 (59%)	3989 (63%)		972 (55%)	1405 (56%)		1497 (62%)	1690 (66%)	
Surgery	147 (4.0%)	107 (3.4%)	0.2	818 (10%)	688 (11%)	0.4	171 (9.7%)	255 (10%)	0.6	255 (11%)	231 (9.1%)	0.089
Radiation	300 (8.2%)	241 (7.6%)	0.3	951 (12%)	777 (12%)	0.8	228 (13%)	313 (13%)	0.7	297 (12%)	293 (12%)	0.4
Chemotherapy	1404 (39%)	1287 (41%)	0.071	2246 (29%)	2038 (32%)	<0.001	465 (26%)	737 (30%)	0.023	671 (28%)	747 (29%)	0.2
Targeted	--	--	0.8	911 (12%)	597 (9.4%)	<0.001	55 (3.1%)	58 (2.3%)	0.11	129 (5.3%)	102 (4.0%)	0.028
Immunotherapy	--	--	0.5	320 (4.1%)	247 (3.9%)	0.6	59 (3.4%)	89 (3.6%)	0.7	52 (2.1%)	50 (2.0%)	0.7

^1^ *n* (%); ^2^ Fisher’s exact test; Pearson’s Chi-squared test; Counts are not presented when fewer than 11 cases were reported within a cell, or where the inclusion of the count would allow for back-calculation of suppressed values. The suppressed cases are included in the total counts for a given category.

## Data Availability

The datasets used to conduct this study are available upon approval of a research protocol from the National Cancer Institute SEER-Medicare Program. Instructions for obtaining these data are available at https://healthcaredelivery.cancer.gov/seermedicare/obtain/ accessed on 22 August 2024.

## Supplementary Materials

The following supporting information can be downloaded at: https://www.mdpi.com/article/10.3390/cancers16172986/s1, Figure S1: Schema of treatment and adverse event selection criteria: the schema depicts the timeline used for adverse event selection for this study; Figure S2: Count of targeted therapies and immunotherapies received by individuals after lung cancer BrM diagnosis; Table S1: Codes used to identify treatment patterns; Table S2: ICD9/10 codes used to identify AEs in the claims data.
