# Supplementary material for: Sex Difference in Disease-Related Adverse Events Post-Diagnosis of Lung Cancer Brain Metastases in Medicare Individuals ≥ 66 Years of Age
Source: Cancers (Basel). 2024 Aug 28;16(17):2986. doi: 10.3390/cancers16172986 (PMC11394199; doi:10.3390/cancers16172986)
Supplement: Supplementary file 1 [file cancers-16-02986-s001.zip › Suppl Table S1.pdf]

Supplementary Table 1. Codes Used to Identify Treatment Patterns.

Table contains treatment codes that were used to identify surgery, radiation and chemotherapies, targeted therapies and immunotherapies.

Treatment Type	Code Classification	Code
Radiation Therapy	ICD-9-CM	92.23–92.24, 92.30–92.33, and 92.39.
	CPT	77261-77263, 77280, 77285, 77290, 77295, 77299–77301, 77305, 77310, 77315, 77321, 77332-77334, 77336-77337, 77370-77372, 77399, 77402-77414, 77416, 77418-77420, 77425, 77427, 77430, 77432, and 0073T.
	HCPCS	G0173-G0174, G0242-G0243, G0251, and G0338-G0340.
Stereotactic radiosurgery	ICD-9-CM	Cranial: 92.30–92.33 and 92.39.
	CPT	Cranial: 61793, 61796-61800, 77371-77372, and 77432.
	HCPCS	Cranial: G0173, G0242, G0243, G0251, G0338, G0339, and G0340.
Neurosurgical resection	ICD-9-CM	01.21–01.25, 01.31, 01.51, and 01.59.
	CPT	61304–61305, 61312–61315, 61320–61321, 61330, 61332–61334, 61340, 61343, 61345, 61440, 61450, 61458, 61460, 61470, 61500–61501, 61510, 61512, 61514, 61516, 61518, 61519- 61522, 61524, 61526, 61530–61531, 61533–61536, 61538- 61539, 61541–61546, 61550, 61552, 61556–61559, 61563- 61564, 61570–61571, 61575–61576, 61580–61586, 61590–61592, 61596–61598, 61600–61601, 61605–61613, and 61615–61616.
Chemotherapy administration services	CPT (Intravenous)	90782-90788, 96408-96419, 96520, 96530, 96545, 96549
	CPT (Not intravenous)	61517, 96400-96406, 96420-96425, 96440-96446, 96450, 96520-96522, 96542, 99601-99602
	HCPCS Level II	Q0083-Q0085, J9000-J9999
	ICD-9 CM (Diagnostic)	V58.1, V58.11, V58.12
**Immunotherapies**		
**Treatment Type +** approval date	**Code Classification**	**Code**
Pembrolizumab 2014	HCPCS	C9027, J9271
	NDC	000063026, 000063029
Nivolumab 2014	HCPCS	C9453, J9299
	NDC	000033734, 000033756, 000033772, 000033774, 000037125
Ipilimumab 2011	HCPCS	C9284, J9228
	NDC	000032327, 000032328
Atezolizumab 2020	HCPCS	C9483, J9022

	NDC	502420917, 50242091701, 50242091786, 502420918, 50242091801, 50242091886
il2	HCPCS	J9015
	NDC	000780495, 654830116
Imiquimod	HCPCS	NA
	NDC	000890610, 000936126, 001151476, 007817152, 293360610, 458020076, 458020368, 548684554, 605050501, 721890084, 992070260
Avelumab	HCPCS	C9491, J9023
	NDC	440873535
Durvalumab	HCPCS	C9492, J9173
	NDC	003104500, 003104611
Cemiplimab	HCPCS	J9113
	NDC	617550008
		**Chemotherapies**
**Treatment Type**	**Code Classification**	**Code**
Carboplatin	HCPCS	J9045
	NDC	00015-3210, 00015-3211, 00015-3212, 00015-3213, 00015-3214, 00015-3215, 00015-3216, 61703-0360, 00703-3249, 47335-0150, 47335-0151, 50742-0447, 50742-0448, 66758-0047, 68083-0190, 68083-0191, 68083-0192, 68083-0193, 71288-0100, 00703-4239, 00703-4244, 00703-4246, 57277-0105, 57277-0106, 67457-0491, 67457-0492, 67457-0493, 67457-0494, 67457-0608, 55150-0386, 16729-0295, 69448-0005, 00703-4248, 61703-0339, 63323-0172, 25021-0202, 47335-0284, 47781-0603, 47781-0604, 47781-0605, 47781-0606, 50742-0445, 50742-0446, 57277-0107
Cisplatin	HCPCS	C9418, J9060, J9062
	NDC	44567-0530, 00015-3070, 00015-3072, 00703-5747, 00703-5748, 16729-0288, 44567-0509, 44567-0510, 44567-0511, 63323-0103, 68001-0283, 68083-0162, 68083-0163, 67457-0424, 67457-0425, 70860-0206, 00069-0081, 00069-0084, 47781-0609, 47781-0610, 61126-0003, 61126-0004
Dacarbazine	HCPCS	C9423, J9130, J9140
	NDC	00703-5075, 63323-0127, 63323-0128, 61703-0327
Docetaxel	HCPCS	J9170, J9171
	NDC	70121-1221, 70121-1222, 70121-1223, 43066-0001, 43066-0006, 43066-0010, 00069-9141, 00069-9142, 00075-8001, 00075-8005, 00409-0366, 00409-0367, 00955-1020, 00955-1021, 00955-1022, 25021-0222, 43598-0258, 43598-0610, 43598-0611, 45963-0765, 63739-0932, 63739-0971, 66758-0050, 66758-0950, 25021-0245, 50742-0431, 50742-0463, 00143-9204, 00143-9205, 43598-0389, 47335-0323, 47335-0895, 47335-0939, 72485-0216, 72485-0215, 72485-0214, 71288-0143, 71288-0144, 71288-0150, 71288-0151, 00409-7870, 00409-0365, 00409-1732, 00409-4235, 00409-5068, 55150-0378, 55150-0379, 55150-0380, 68083-0401, 68083-0400, 68083-0399, 00409-0201, 00409-0368, 16729-0231, 16729-0267, 43598-0259, 45963-0734, 47335-0285, 67457-0533, 67457-0781, 69097-0372, 70700-0174, 70700-0175, 70700-0176, 00075-8003, 00075-8004, 00703-5720, 00703-5730, 16714-0465, 16714-0500, 16729-0120, 16729-0228, 39822-2120, 39822-2180, 39822-2200,

		42367-0121, 45963-0781, 45963-0790, 50742-0428, 00069-9144, 00409-0369, 67457-0531, 67457-0532, 69097-0369, 69097-0371, 57884-3021
Etoposide	HCPCS	C9414, C9425, J8560, J9181, J9182
	NDC	00378-3266, 00703-5653, 55390-0291, 55390-0292, 55390-0293, 55390-0491, 55390-0492, 55390-0493, 63323-0104, 00703-5657, 00703-5656, 16729-0114, 68001-0265, 00015-3404, 16729-0262
Gemcitabine	HCPCS	J9201, J9198
	NDC	68001-0359, 68001-0350, 68001-0348, 68001-0342, 16714-0909, 16714-0930, 16729-0391, 16729-0419, 16729-0423, 00703-5775, 00703-5778, 00002-7502, 00781-3282, 00781-3283, 16729-0092, 16729-0117, 16729-0118, 55390-0391, 67457-0462, 67457-0463, 67457-0464, 68001-0282, 68083-0148, 68083-0149, 69097-0313, 69097-0314, 00409-0181, 00409-0182, 00409-0183, 00409-0185, 00409-0187, 25021-0209, 25021-0234, 25021-0235, 42236-0001, 42236-0002, 55111-0686, 55111-0687, 70860-0204, 70860-0205, 63323-0102, 63759-3028, 63759-3029, 71288-0117, 72485-0221, 72485-0222, 72485-0223, 00002-7501, 00143-9394, 00143-9395, 00409-0186, 23155-0213, 23155-0528, 25021-0208, 45963-0619, 63323-0125, 63323-0126, 67457-0616, 67457-0617, 67457-0618, 00069-3857, 00069-3858, 00069-3859, 00591-3562, 00591-3563, 23155-0214, 23155-0483, 23155-0484, 23155-0529, 25021-0239, 45963-0612, 45963-0620, 45963-0623, 45963-0624, 45963-0636, 47335-0153, 47335-0154, 50742-0496, 50742-0497, 50742-0498, 16729-0426, 62756-0008, 62756-0073, 62756-0102, 62756-0219, 62756-0321, 62756-0438, 62756-0533, 62756-0614, 62756-0746, 62756-0974, 71288-0113, 71288-0114, 60505-6113, 60505-6114, 60505-6115
Irinotecan	HCPCS	C9474, J9206
	NDC	66758-0048, 68001-0284, 69171-0398, 68001-0480, 68083-0381, 70700-0169, 45963-0614, 55150-0352, 55150-0353, 55150-0354, 68001-0425, 68001-0426, 72485-0213, 68083-0382, 70700-0170, 00009-1111, 00009-7529, 00143-9583, 00143-9701, 00143-9702, 00703-4432, 00703-4434, 15054-0043, 16714-0027, 16714-0131, 25021-0214, 25021-0230, 47335-0937, 47335-0953, 50742-0401, 50742-0402, 59923-0702, 59923-0714, 59923-0715, 59923-0716, 61703-0349, 63323-0193, 72485-0211, 72485-0212, 16714-0725, 16714-0726, 23155-0179
Lomustine	HCPCS	S0178
	NDC	00015-3030, 00015-3031, 00015-3032, 58181-3030, 58181-3031, 58181-3032, 58181-3040, 58181-3041, 58181-3042, 58181-3043
Lurbinectedin	HCPCS	J9223
	NDC	687270712,
Paclitaxel	HCPCS	C9127, C9431, J9264, J9265, J9267
	NDC	47781-0595, 55390-0114, 55390-0304, 55390-0314, 66758-0043, 67457-0434, 67457-0449, 67457-0471, 68083-0178, 68083-0179, 68083-0180, 68817-0134, 70860-0200, 00703-3216, 00703-3217, 00703-3213, 00703-3218, 16714-0137, 69539-0158, 69539-0159, 69539-0157, 72205-0063, 72205-0062, 72205-0061, 00703-4764, 00703-4768, 44567-0504, 44567-0505, 44567-0506, 45963-0613, 61703-0342, 63323-0763, 00069-0076, 00069-0078, 00069-0079, 00703-4766, 00703-4767, 25021-0213, 68001-0516, 47781-0593, 47781-0594, 51991-0937, 51991-0938, 70860-0215
Pemetrexed	HCPCS	C9213, J9304, J9305
	NDC	00002-7623, 00002-7640, 67184-0503

	HCPCS	C1086, C9253, J8700, J9328
Temozolomide	NDC	54868-4142, 54868-5348, 54868-5350, 54868-5354, 54868-5980, 62175-0240, 62175-0241, 62175-0242, 62175-0243, 62175-0244, 62175-0245, 64144-0501, 64144-0502, 64144-0503, 64144-0504, 64144-0505, 64144-0506, 64980-0333, 64980-0334, 64980-0335, 64980-0336, 64980-0337, 64980-0338, 65162-0801, 65162-0802, 65162-0803, 65162-0804, 65162-0805, 65162-0806, 67877-0537, 67877-0538, 67877-0539, 67877-0540, 67877-0541, 67877-0542, 69189-7638, 00085-0381, 16729-0048, 16729-0050, 16729-0051, 16729-0129, 16729-0130, 40051-0604, 40051-0605, 40051-0606, 40051-0607, 40051-0608, 40051-0609, 47335-0893, 00054-0320, 00054-0321, 00054-0322, 00054-0323, 00054-0324, 00054-0325, 16729-0049, 50268-0761, 50268-0762, 47335-0890, 47335-0891, 47335-0892, 47335-0929, 47335-0930, 62559-0921, 62559-0920, 62559-0922, 62559-0923, 62559-0924, 62559-0925, 00085-3004, 00085-1366, 00085-1381, 00085-1417, 00085-1425, 00085-1430, 00085-1519, 00093-7599, 00093-7600, 00093-7601, 00093-7602, 00093-7638, 00093-7639, 00378-5260, 00378-5261, 00378-5262, 00378-5263, 00378-5264, 00378-5265, 00527-1777, 00527-1778, 00527-1779, 00527-1780, 00527-1781, 00527-1782, 00781-2691, 00781-2692, 00781-2693, 00781-2694, 00781-2695, 00781-2696, 42737-0101, 42737-0102, 42737-0103, 42737-0104, 42737-0105, 42737-0106, 43975-0252, 43975-0253, 43975-0254, 43975-0255, 43975-0257, 50268-0763, 51862-0083, 51862-0084, 51862-0085, 51862-0086, 51862-0087, 51862-0088, 75834-0132, 75834-0142, 75834-0143, 75834-0144, 75834-0145, 43975-0256, 59923-0703, 59923-0704, 59923-0705, 59923-0706, 59923-0707, 59923-0708, 59923-0709, 59923-0710, 59923-0711, 59923-0712, 59923-0713,
	HCPCS	J8705, J9350, J9351
Topotecan	NDC	66758-0051, 66435-0410, 67457-0474, 00007-4201, 00007-4205, 00007-4207, 00069-0075, 00078-0672, 00078-0673, 00078-0674, 00409-0302, 00703-4714, 16729-0151, 16729-0243, 45963-0615, 55390-0370, 62756-0023, 63323-0762, 67457-0662, 71288-0127, 25021-0206, 25021-0236, 25021-0824, 50742-0404
	HCPCS	C9440, J9390
Vinorelbine	NDC	55390-0069, 55390-0070, 00008-0045, 64370-0532, 61703-0341, 00069-0099, 00069-0103, 00069-0205, 00703-4182, 00703-4183, 25021-0204, 45963-0607, 66758-0045, 67457-0431, 67457-0479, 67457-0481, 67457-0482, 50742-0420, 50742-0427
Carmustine	HCPCS	628560177, 007813474, 712880126, 435980628, 167290548, 167290545, 708600223, 548790036, 684750503, 701211482, 707101525, 507420492, 243380050, 231550790, 231550649, 000153012, 231550261
	NDC	C9437, J9050
Methotrexate	HCPCS	J8610, J9250, J9260, J9255
	NDC	007033673,007033678,009046012,167290277,512850366,512850367,512850368,512850369,545691818, 633230122,633230123,674570465,000690147,000690148,000690149,000690181,000690204,216950111, 422540110,422910594,430630439,500900294,500902345,548680173, 552890924,422910505,633230121,663360338,667580040,667580041,672530320,674570221,674570466, 674570467,674570480,674570485,674570486,683820775,705180223,705181251,705182711,758400111,

		713351772,617030350,617030408,705183263,636291472,707711058,617030124,548684716,553900031,553900032,617030351,617030352,705183672,007033671,007033675,101390062,553900033,553900034,621350772,705183777,713352221,553900143,477810482,502680527,000544550,000548550,510790670,819270204,167290486,526522001,596510182,705181398,003780014,005550572,001439830,692381423,001439516,001439517,001439518,617860417,000690146,493490314,493490406,548683826

**Targeted therapies**		
**Treatment Type**	**Code Classification**	**Code**
Dabrafenib	HCPCS	NA
	NDC	000780681, 00078068166, 000780682, 00078068266, 001730846, 00173084608, 001730847, 00173084708, 00173084761, 00173084765, 538730023, 53873002300, 538730024, 53873002400, 53873002401
Trametinib	HCPCS	NA
	NDC	120640015, 12064001500, 120640016, 12064001600, 120640017, 12064001700, 000780666, 00078066615, 000780668, 00078066815, 001730848, 00173084813, 00173084865, 001730849, 00173084913, 001730858, 00173085813, 73309004101, 73309021101, 73309021102, 73309021103, 00078111294, 000781105, 000781112, 00078110515, 00078111215
Dasatinib	HCPCS	NA
	NDC	62157077601, 62157087401, 00002052711, 000030524, 00003052411, 000030527, 00003052711, 000030528, 00003052811, 000030852, 00003085211, 00003085222, 000030855, 00003085522, 000030857, 00003085722, 73309002901, 548685759, 54868575900, 82922001201, 86032000801
Imatinib	HCPCS	S0088
	NDC	NA
brigatinib	HCPCS	NA
	NDC	761890113
lorlatinib	HCPCS	NA
	NDC	000690227, 000690231, 635390927
afatinib	HCPCS	NA
	NDC	005970137, 005970138, 005970141
dacomitinib	HCPCS	NA
	NDC	000690197, 000691198, 000692299, 635390197
cetuximab	HCPCS	C9215, J9055
	NDC	667330948, 667330958
