# Supplementary material for: Sex Difference in Disease-Related Adverse Events Post-Diagnosis of Lung Cancer Brain Metastases in Medicare Individuals ≥ 66 Years of Age
Source: Cancers (Basel). 2024 Aug 28;16(17):2986. doi: 10.3390/cancers16172986 (PMC11394199; doi:10.3390/cancers16172986)
Supplement: Supplementary file 1 [file cancers-16-02986-s001.zip › Suppl Table S2.pdf]

Supplemental Table 2. ICD9/10 codes used to identify AEs in the claims data.

AE	ICD9	ICD10
Epilepsy	345.xx, 780.3x	G40.xx, R56.xx
Ischemic Stroke	433.x, 434.x, 435.x, 436.x	I63
Hemorrhagic Stroke	430,431,432.x	I60, I61
Vasogenic Edema	348.5	G93.6
Brain Herniation	348.4	G93.5
Hydrocephalus	331.4	G91
Neurological Deficit	349	R29
Headaches	784	R51
Vision Difficulty	360-379	H53
Paralyses	728	M62
Leptomeningeal Disease	C79.49x, C80.1x, G96.198, R90.89x	198.4x, 199.1x, 322.9x,794.09x
