# Supplementary figures and images for: Sex Difference in Disease-Related Adverse Events Post-Diagnosis of Lung Cancer Brain Metastases in Medicare Individuals ≥ 66 Years of Age
Source: Cancers (Basel). 2024 Aug 28;16(17):2986. doi: 10.3390/cancers16172986 (PMC11394199; doi:10.3390/cancers16172986)

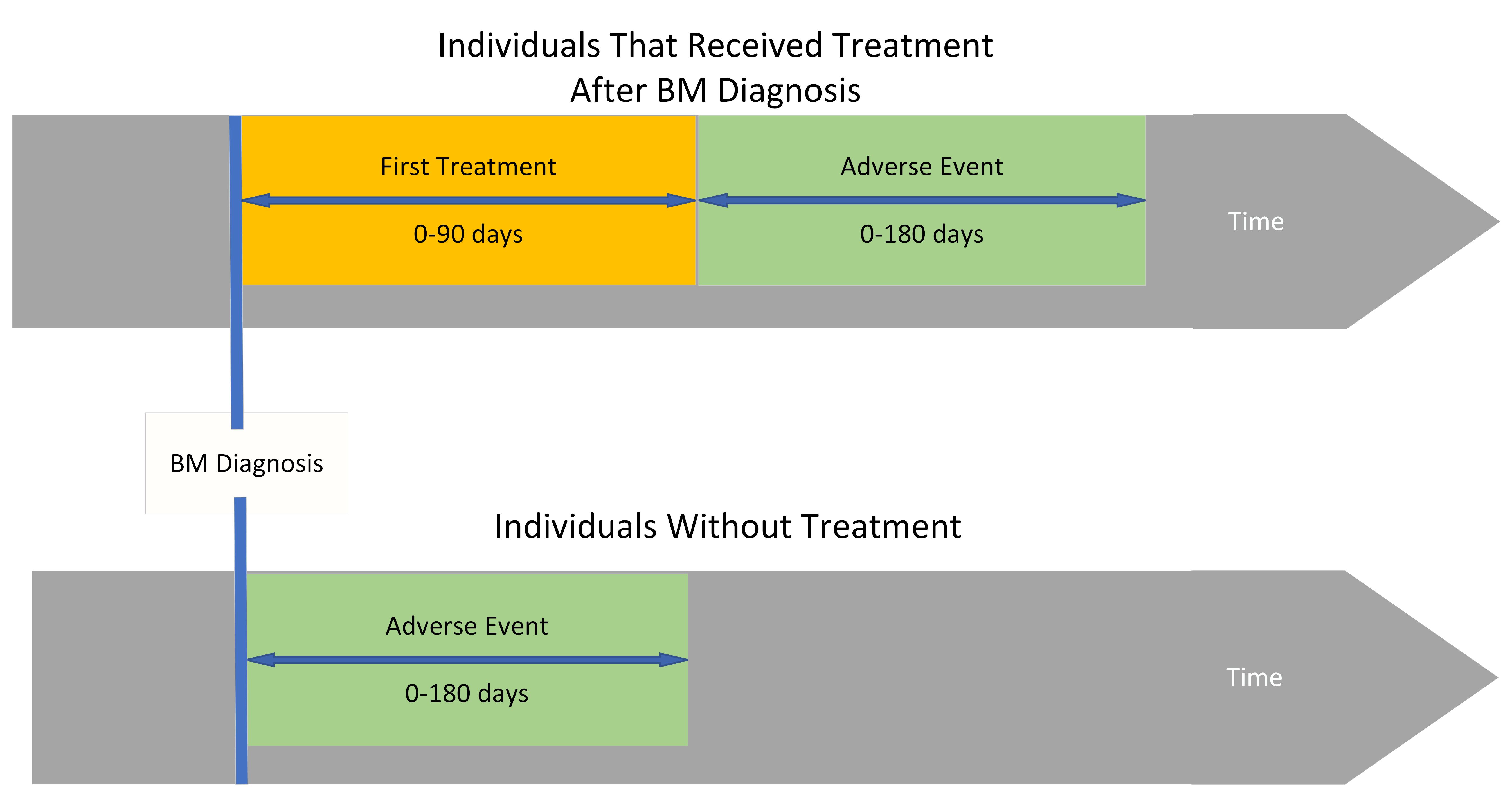

Supplement: Supplementary file 1 [file cancers-16-02986-s001.zip › Suppl Figure S1 .png]

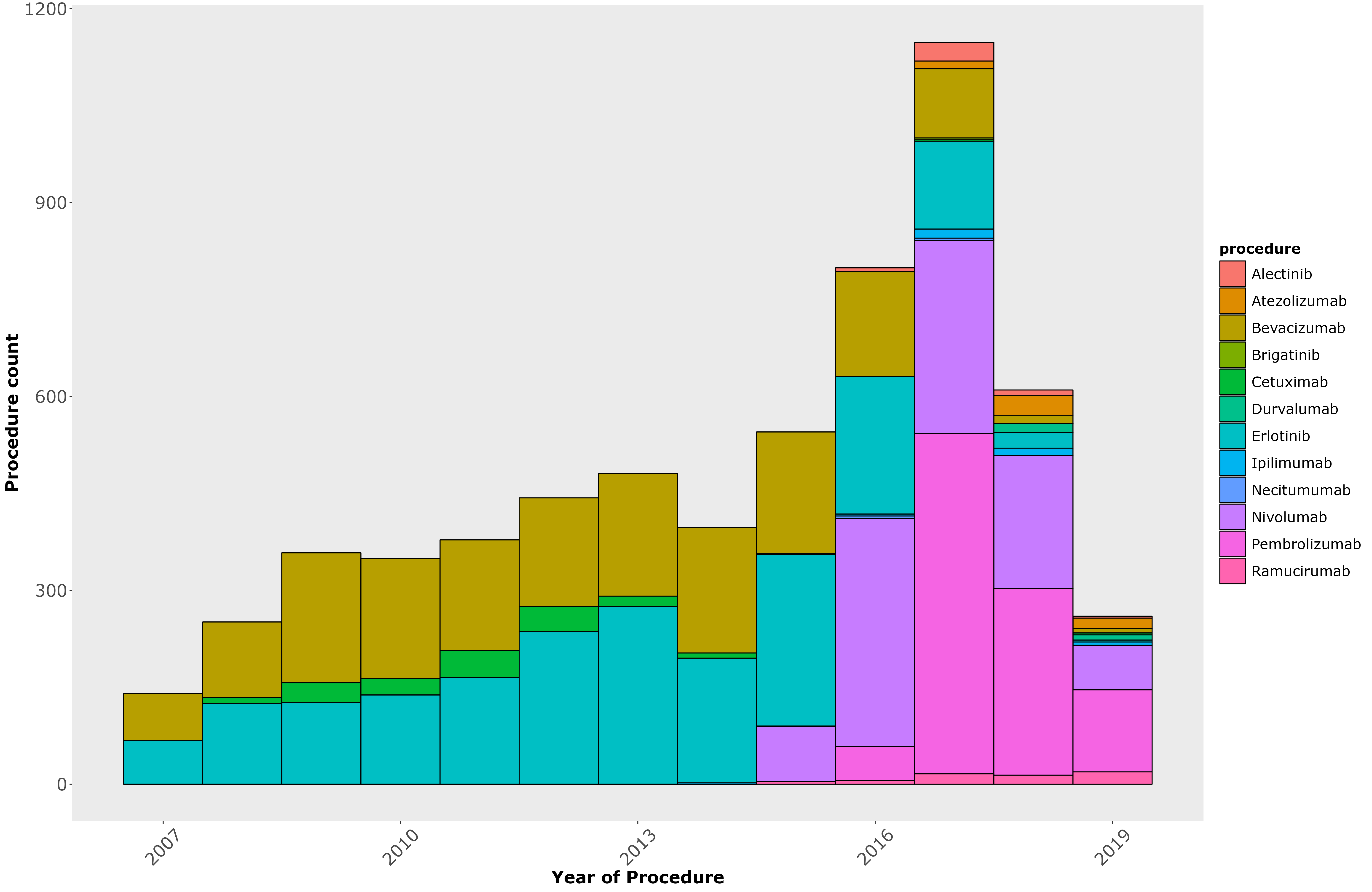

Supplement: Supplementary file 1 [file cancers-16-02986-s001.zip › Suppl Figure S2.png]
